# A Community-based study on the prevalence and predisposing factors of Parkinson’s disease in Barangay Mangilag Sur, Quezon Province, Philippines

**DOI:** 10.1016/j.prdoa.2022.100169

**Published:** 2022-10-26

**Authors:** Raymond L. Rosales, Mary Camille E. Rosales, Danica Jane S.J. Robles, Ron Christian Neil T. Rodriguez, Nadia Beatrice S. Romana, Joseph Mariuz B. Rosales, Gerardo B. Salazar, Richelle Ann S. Santiano

**Affiliations:** a Faculty of Medicine and Surgery, University of Santo Tomas, Manila, Philippines; b University of Santo Tomas Hospital, Espana, Manila, Philippines; c Center for Neuro-diagnostic and Therapeutic Services, Metropolitan Medical Center, Manila, Philippines; d Institute for Neurosciences, Saint Luke’s Medical Centre, Quezon City, Philippines; e Lucena Doctors Medical Center, Quezon Province, Philippines

**Keywords:** Parkinson’s disease, Prevalence, Risk Factors, Community Survey

## Abstract

**Introduction:**

Prevalence of Parkinson’s Disease (PD) in the Philippines has been estimated to be < 1 % based on a 2007 nationwide survey conducted by the Philippine Neurological Association, but without case ascertainment. Since there is still paucity of data, we aim to determine the prevalence of PD in a rural community and the possible predisposing factors on the development of the disease.

**Methods:**

This is a two-phase descriptive study which investigated the prevalence of PD and associated risk factors in a randomly selected rural community in Candelaria, Quezon Province. A validated screening questionnaire was utilized, and case ascertainment was done in eligible respondents.

**Results:**

A total of 365 respondents aged ≥ 20 years were randomly surveyed from 2016 to 2017. Two cases of PD aged ≥ 60 years were reported. Thus, the prevalence of PD in the community was 0.55 %. Age-specific prevalence of PD among individuals ≥ 60 years was 4.35 %.

Insecticide use was infrequent in the community and was recorded in one PD patient. Protective factors like smoking and drinking tea were not observed in both cases whereas coffee intake was reported in one PD patient.

**Conclusion:**

This community-based epidemiologic study on PD is consistent with a nationwide study. The study portrayed certain demographic and environmental features inherent in a community, which are potential confounding variables in PD development. Future larger population studies be recommended to establish PD in the advancing age and to further support the link of various factors with PD.

## Highlights

• The low prevalence of Parkinson’s disease in the rural setting is consistent with a nationwide study.
• PD cases were reported among older adults.
• Some demographic and environment features which can contribute in developing PD were noted in the community.

## 1 Introduction

Parkinson’s Disease (PD) is a neurodegenerative disorder characterized by bradykinesia, resting tremors, rigidity, postural instability, and non-motor features such as autonomic, sensory, neuropsychiatric and sleep disorders. The number of individuals affected by PD leading to disability is expected to go beyond 12 million by 2040 [1].

Aside from advancing age, environmental factors that may increase the risk of having PD include pesticides, herbicides and heavy metals [2]. Conversely, tobacco, coffee and tea, non-steroidal anti-inflammatory drugs [2], [3], urate, calcium channel blockers [3], antioxidants, vitamin B6, unsaturated fatty acids, and physical activity are found to reduce the risk of PD [2]. Possible link with PD are also observed with head trauma, olfactory impairment, risk-avoiding personality, depression and anxiety [2]. The association between PD and other factors such as iron and estrogen [2], [3], alcohol, cancer, farming, living in rural areas, drinking well water [2], and metabolic risk factors [3] are still uncertain.

Genetic factors are also studied in rare familial forms of PD. Mutations in LRRK2 gene is the most frequent worldwide, then PINK1 mutations are implicated in autosomal recessive early-onset PD in Filipinos [4]. In recent years, the Philippines has become a trajectory for research in heredodegenerative movement disorders, specifically X-linked Dystonia-Parkinsonism (XDP) [5], believed to be the model disease in understanding the movement disorder construct [6]. This disease is commonly seen in the country’s Panay Island.

Although half of the world’s aging population is in Asia, the prevalence of PD is still lower compared to Western countries [4], [7]. Prevalence studies have always been a challenge in the country despite the estimated population being around 110 million people (https://popcom.gov.ph/), because it is an archipelago with>7,000 islands. A community-based prevalence study of PD conducted in 2007 by the Philippine Neurological Association (PNA) revealed that the prevalence of parkinsonism was < 1 % [8]. However, the study fell short of case ascertainment by specialists [8]. A recent study in two movement specialty centers in Metro Manila from 2007 to 2019 showed that PD is still the most common movement disorder (n = 606, 42.1 %) [9].

In terms of prevalence of PD, much is still unknown, especially from marginalized or rural communities. With this in mind, the study aims to determine the prevalence of PD in the non-urbanized community setting and enumerate risk factors that predispose to the development of PD.

## 2 Methods

This is a descriptive study which used a two-phase design to assess the prevalence and risk factors of PD in a rural community. Systematic randomization of regions, provinces, districts, municipalities and barangays (smallest political division) in Luzon was done. Barangay Mangilag Sur in Candelaria, Quezon was chosen. The Visayas and Mindanao islands were excluded due to the prevalence of XDP in those regions.

Using the updated 2010 census of Barangay Mangilag Sur, there were 1,406 households in total, with 6,706 individuals meeting the inclusion criteria. Sample size of 365 individuals was included in the study. The target population was randomly selected and consisted of eligible residents who are aged ≥ 20 years and permanent residents and/or residents of nursing homes in the community. Individuals were excluded if they had died or if they no longer resided in the community. Meanwhile, a dedicated training on how to clinically examine a patient was undertaken by the researchers, under the guidance of a trained specialist.

A pilot study was conducted to determine if the questionnaire was comprehensible for the respondents. In Phase 1, the researchers visited homes in the barangay to administer the questionnaire which encompassed demographic profile, past medical, lifestyle, and family history. It also consisted of two questions (presence of tremors and/or bradykinesia) (Supplementary Table 1) that had been validated by PNA [10] and a third question of previous diagnosis of PD. If the participant is unable to answer the questions due to speech or cognitive problems, a caregiver may respond on the participant’s behalf.

**Table 1:** Correlation of demographic, environmental factors and associated symptoms with Parkinson’s Disease (PD).

**Parameter**	**Rest Tremors**	**Bradykinesia**	**Diagnosed with PD**
	**n = 6**	**n = 2**	**n = 2**
**Age Group**			
**20**–**29 years**	0.002	–	–
**30**–**39 years**	0.002	–	–
**40**–**49 years**	–	–	–
**50**–**59 years**	0.003	–	–
**>60 years**	0.011	0.022	0.022
**Sex**			
**Male**	0.003	0.003	0.003
**Female**	0.003	0.003	0.003
**Water Source**			
**Tap Water**	0.002	0.002	0.002
**Deep Well**	0.005	0.007	0.007
**Coffee Intake**			
**No**	0.004	0.006	0.006
**Yes (cups/day)**	0.004	0.003	0.003
**0**–**1**	0.006	0.006	0.006
**######**	0.002	–	–
**>3**	–	–	–
**Tea Intake**			
**No**	0.003	0.003	0.003
**Yes (cups/day)**	0.003	–	–
**0**–**1**	–	–	–
**######**	0.033	–	–
**>3**	–	–	–
**Alcohol Intake**			
**No**	0.004	0.005	0.005
**Yes (bottles/session)**	0.001	–	–
**0**–**1**	0.001	–	–
**######**	–	–	–
**>3**	–	–	–
**Smoking**			
**No**	0.003	0.003	0.003
**Yes (sticks/day)**	–	–	–
**1**	–	–	–
**######**	–	–	–
**>3**	–	–	–
**Insecticide Usage**			
**Occupational**	–	–	–
**Home Use**	0.002	0.004	0.004
**Not Used**	0.003	0.002	0.002
**Associated Symptoms of PD**			
**Anxiety**	0.013	0.013	0.013
**Depression**	0.025	0.05	0.05
**Easy Fatigability**	0.02	0.029	0.029
**Insomnia**	0.016	0.016	0.016
**Diarrhea**	–	–	–
**Pain**	0.056	–	–

Pearson r value of 0.001 to 0.190 will be considered as very weak correlation; 0.20 to 0.39 as weak correlation; 0.400 to 0.590 as moderate correlation; 0.600 to 790 as strong correlation; 0.800 to 1 as very strong correlation.

Participants who responded positively to any of the 3 questions were considered eligible for Phase 2 evaluation. The potential cases underwent videotaping following an informed and written consent. The video was reviewed by a movement disorder subspecialist (“parkinsonologist”) for screening.

In Phase 2 or case ascertainment phase, a local neurologist and a parkinsonologist confirmed the diagnosis of PD among the suspected cases. This established the point prevalence of the condition in the given locale.

### 2.1 Statistical analysis

Statistical analyses were performed using Statistical Package for Social Services V 26. Descriptive statistics included mean, median, frequency and percentage. To determine the association of demographic, environmental and hereditary factors with the occurrence of rest tremors, bradykinesia and PD, Pearson correlation coefficient was utilized.

### 2.2 Ethics

The study was conducted according to the guidelines of Declaration of Helsinki, and approved by the Institutional Review Board of Metropolitan Medical Center. Initial protocol approval was provided by the Department of Clinical Epidemiology, Faculty of Medicine and Surgery, University of Santo Tomas.

Further details of the methodology and conduct of the survey have been published earlier [11]. Part of this study was poster-presented during the annual convention of the International Parkinson’s and Movement Disorders Society held at Nice, France in 2019.

## 3 Results

### 3.1 Community profile

Three hundred sixty-five respondents from the five areas of Barangay Mangilag Sur were randomly surveyed from 2016 to 2017. The study population was evenly distributed between males (49.86 %) and females (50.14 %), with majority being within the age range of 20–29 years old (30.14 %), followed by those between 30 and 39 years old (22.47 %). Most of the respondents had part-time jobs or were students (‘Others’) accounting for 46.03 % of the study population, and had an educational attainment of high school level (39.18 %). Majority of the respondents were also Catholics (69.86 %), married (66.85 %) (Supplementary Table 2), and earn a monthly salary of<10,000 pesos (193.04 USD) (53.42 %).

### 3.2 Prevalence of Parkinson’s disease in the community

Six respondents presented with rest tremors (1.64 %). Two of whom, aged ≥ 60 years (male, 61 years and female, 62 years) also had asymmetric bradykinesia (0.55 %) (Supplementary Table 3). These latter individuals were confirmed to have PD, and were assessed to be on their early stages, given that retropulsion test did not result in falling too. Thus, the prevalence of PD in Barangay Mangilag Sur is 0.55 %, whereas, age-specific prevalence of PD among individuals ≥ 60 years is 4.35 %. Notably, the four other cases with resting tremor, unrelated and coming from separate families, had symmetrical presentation. Kinetic tremors and cerebellar signs were present in two of those cases, pointing to possible ataxia syndromes. Atypical parkinsonism was the possibility for the remaining two of four cases.

### 3.3 Risk factors and associated symptoms of Parkinson’s disease present in the community

Insecticide usage is the most frequent factor associated with PD, and was recorded in one of the PD respondents who use it at home. One of the two gets water from deep well. Obtaining water from deep well which is unregulated by health authorities may be contaminated by chemicals that may elevate the risk of having PD. In terms of protective factors, most of the individuals including one PD respondent, are coffee drinkers, consuming at least a cup per day, but neither of the two drink tea. Smoking and alcohol drinking were not reported in both cases. Head trauma which may be a potential risk factor was not documented in both cases. Family history of PD was also not present in the PD respondents. Depression and easy fatigability were recorded in both cases while anxiety and insomnia were noted in one of the two.

Very weak correlation was found between the following factors: age, sex, source of water, coffee, tea or alcohol intake, smoking, insecticide usage, and associated symptoms; and PD and related motor symptoms (Table 1).

## 4 Discussion

The gold standard for prevalence studies of PD remains to be a survey with direct neurologic examination performed by a subspecialist. However, this method requires more resources in terms of time, effort, and funds. Our study was able to capture certain demographic and local features inherent in a community and perform case ascertainment. Considering both sexes, the prevalence of PD increased with advancing age. Asymmetrical initial presentation is usual in PD, and this makes the clinical diagnosis of PD robust. This was observed in both cases but was initially disregarded by the individuals, until they noticed gradual slowing of movement and rigidity. Since they were still able to perform daily activities, they did not seek consult and receive treatment. Interestingly, an Asian perspective alluded to a potential mismatch between the clinician’s view and the patient’s view of what aspects of PD mostly affect their daily lives [12]. Also, due to the similarity of the symptoms of PD with other health disorders, it has been poorly recognized and underdiagnosed in the Philippines. Furthermore, due to the geography of the Philippine islands, it is difficult for neurologists to confirm reported cases of PD as some areas are inaccessible. This strengthens the importance of case-ascertained studies in obtaining reliable information on the prevalence of this condition.

Even though motor symptoms of PD are the most significant abnormality in PD, non-motor features may occur in the early and advanced stages of the disease, and carry an impact on the individual’s quality of life. For instance, the occurrence of depression [13], [14], anxiety [13] and fatigue [15] in Filipinos diagnosed with PD have been well documented in local cohort studies, as well as in this study.

Since some of the risk factors involved in PD are modifiable, lifestyle change is still vital in approaching PD. The very weak correlation shown between the presenting demographic and environmental factors and occurrence of rest tremors, bradykinesia and PD could be attributed to the low prevalence of PD in the community. A larger population size and inclusion of other factors such as medications and physical activity of the respondents may be recommended to determine a definite association of abovementioned factors with PD.

In conclusion, the prevalence of PD in the locale (0.55 %) is consistent with the nationwide prevalence of PD in the country (<1%) [8]. Through this study, formulation of programs that could improve the awareness and primary health care of Filipinos with PD can be explored. The collected data on the prevalence of PD can also open opportunities for establishment of the country’s standardized database for movement disorders and future prospective studies. This can also be a trigger to develop more epidemiologic studies to accurately depict the prevalence of PD in urban and/or rural regions as well as in the whole country.

## CRediT authorship contribution statement

**Raymond L. Rosales:** Conceptualization, Methodology, Investigation, Formal analysis, Writing – review & editing, Supervision, Resources. **Mary Camille E. Rosales:** Conceptualization, Methodology, Investigation, Data curation, Writing – original draft, Formal analysis, Writing – review & editing. **Danica Jane S.J. Robles:** Conceptualization, Methodology, Investigation, Data curation, Writing – original draft. **Ron Christian Neil T. Rodriguez:** Conceptualization, Methodology, Investigation, Data curation, Writing – original draft. **Nadia Beatrice S. Romana:** Conceptualization, Methodology, Investigation, Data curation, Writing – original draft. **Joseph Mariuz B. Rosales:** Conceptualization, Methodology, Investigation, Data curation, Writing – original draft. **Gerardo B. Salazar:** Investigation, Supervision, Resources. **Richelle Ann S. Santiano:** Formal analysis, Writing – review & editing.

## Declaration of Competing Interest

The authors declare that they have no known competing financial interests or personal relationships that could have appeared to influence the work reported in this paper.

## Data Availability

The raw data that support the results of this study are available from the corresponding author on reasonable request. Funding This research did not receive any specific grant from funding agencies in the public, commercial, or not-for-profit sectors.
