# Supplementary material for: A Community-based study on the prevalence and predisposing factors of Parkinson’s disease in Barangay Mangilag Sur, Quezon Province, Philippines
Source: Clin Park Relat Disord. 2022 Oct 26;7:100169. doi: 10.1016/j.prdoa.2022.100169 (PMC9637797; doi:10.1016/j.prdoa.2022.100169)
Supplement: Supplementary data 1 [file mmc1.docx]

**Supplementary Table 1.** Modified from PNA Questionnaire with sensitivities and specificities focusing on Parkinsonism [10]

| Illness | Questions (any history of…) | Sensitivity | Specificity |
| --- | --- | --- | --- |
| Parkinsonism | Several months of having HAND TREMORS while at rest | 0.586 (0.469-0.694) | 0.891 (0.870-0.910) |
|  | Several months of SLOWNESS in movement or moving like a “ROBOT” | 0.725 (0.610-0.816) | 0.853 (0.829-0.874) |
|  | Question 5 OR 6 | 0.771 (0.660-0.854) | 0.801 (0.774-0.825) |

**Supplementary Table 2.** Demographic, environmental, and medical profile of the respondents

| Parameter | Number of Respondents (N=365) | Percentage of Study Population |
| --- | --- | --- |
| Age Group |  |  |
| 20-29 years  30-39 years  40-49 years  50-59 years  ≥ 60 years | 110  82  70  57  46 | 30.14%  22.47%  19.18%  15.62%  12.60% |
| Sex |  |  |
| Male  Female | 182  183 | 49.86%  50.14% |
| Occupation |  |  |
| Professional  Clerical  Others  Unemployed | 44  37  168  116 | 12.05%  10.14%  46.03%  31.78% |
| Religion |  |  |
| Catholic  Christian  Iglesia ni Cristo  Islam  Others | 255  102  8  0  0 | 69.86%  27.95%  2.19%  0%  0% |
| Educational Attainment |  |  |
| Elementary  High School  College  N/A | 73  143  105  44 | 20%  39.18%  28.77%  12.05% |
| Civil Status |  |  |
| Single  Married  Separated  Widowed | 97  244  5  19 | 26.58%  66.85%  1.37%  5.21% |
| Residence |  |  |
| Owned  Rented | 285  80 | 78.08%  21.92% |
| Water Source |  |  |
| Tap Water  Deep Well | 292  73 | 80%  20% |
| Food Source |  |  |
| Homegrown  Market | 21  344 | 5.75%  94.25% |
| Garbage Disposal |  |  |
| Garbage Collector  Own (times/week)  1  2-3  >3 | 17  348  134  81  133 | 4.66%  95.34%  36.71%  22.19%  36.44% |
| Coffee Intake |  |  |
| No  Yes (cups/day)  0-1  2-3  >3 | 83  282  184  83  15 | 22.74%  77.26%  50.41%  22.74%  4.11% |
| Tea Intake |  |  |
| No  Yes (cups/day)  0-1  2-3  >3 | 308  57  52  5  0 | 84.38%  15.62%  14.25%  1.37%  0% |
| Alcohol Intake |  |  |
| No  Yes (bottles/session)  0-1  2-3  >3 | 200  165  131  18  16 | 54.79%  45.21%  35.89%  4.93%  4.38% |
| Smoking |  |  |
| No  Yes (sticks/day)  1  2-3  >3 | 290  75  17  6  52 | 79.45%  20.55%  4.66%  1.64%  14.25% |
| Insecticide Usage |  |  |
| Occupational  Home Use  Not Used | 2  137  226 | 0.55%  37.53%  61.92% |
| Associated Symptoms of Parkinson’s Disease |  |  |
| Anxiety  Depression  Easy Fatigability  Insomnia  Diarrhea  Pain | 38  20  34  32  4  3 | 10.41%  5.48%  9.32%  8.77%  1.10%  0.82% |
| Past Medical History |  |  |
| Respiratory  Cardiovascular  Nephrologic  Endocrine  Musculoskeletal  Hematologic  Oncologic  Infectious  Neurologic  Gastrointestinal  Others | 19  35  1  9  5  1  1  5  4  3  1 | 5.21%  9.59%  0.27%  2.47%  1.37%  0.27%  0.27%  1.37%  1.10%  0.82%  0.27% |
| Family Illnesses |  |  |
| Parkinson’s Disease  Hypertension  Diabetes Mellitus  Stroke  Cancer  Others | 2  95  40  29  8  103 | 0.55%  26.03%  10.96%  7.95%  2.19%  28.22% |

**Supplementary Table 3.** Prevalence of rest tremors and bradykinesia per age group

| Age Group | Total Population | Rest Tremors | Percentage from Age Group Population | Bradykinesia | Percentage from Age Group Population |
| --- | --- | --- | --- | --- | --- |
| 20-29  30-39  40-49  50-59  ≥60 | 110  82  70  57  46 | 1  1  0  1  3 | 0.91%  1.22%  0%  1.75%  6.52% | 0  0  0  0  2 | 0%  0%  0%  0%  4.35% |
| Total | 365 | 6 | 1.64% | 2 | 0.55% |
